# Expanded ataxin-7 cause toxicity by inducing ROS production from NADPH oxidase complexes in a stable inducible Spinocerebellar ataxia type 7 (SCA7) model

**DOI:** 10.1186/1471-2202-13-86

**Published:** 2012-07-24

**Authors:** Abiodun Ajayi, Xin Yu, Staffan Lindberg, Ülo Langel, Anna-Lena Ström

**Affiliations:** 1 Department of Neurochemistry, Stockholm University, Svante Arrhenius väg 21A, SE-106 91, Stockholm, Sweden

**Keywords:** Ataxin-7, NADPH oxidase complex, Neurodegeneration, Oxidative stress, Polyglutamine, SCA7

## Abstract

**Background:**

Spinocerebellar ataxia type 7 (SCA7) is one of nine inherited neurodegenerative disorders caused by polyglutamine (polyQ) expansions. Common mechanisms of disease pathogenesis suggested for polyQ disorders include aggregation of the polyQ protein and induction of oxidative stress. However, the exact mechanism(s) of toxicity is still unclear.

**Results:**

In this study we show that expression of polyQ expanded ATXN7 in a novel stable inducible cell model first results in a concomitant increase in ROS levels and aggregation of the disease protein and later cellular toxicity. The increase in ROS could be completely prevented by inhibition of NADPH oxidase (NOX) complexes suggesting that ATXN7 directly or indirectly causes oxidative stress by increasing superoxide anion production from these complexes. Moreover, we could observe that induction of mutant ATXN7 leads to a decrease in the levels of catalase, a key enzyme in detoxifying hydrogen peroxide produced from dismutation of superoxide anions. This could also contribute to the generation of oxidative stress. Most importantly, we found that treatment with a general anti-oxidant or inhibitors of NOX complexes reduced both the aggregation and toxicity of mutant ATXN7. In contrast, ATXN7 aggregation was aggravated by treatments promoting oxidative stress.

**Conclusion:**

Our results demonstrates that oxidative stress contributes to ATXN7 aggregation as well as toxicity and show that anti-oxidants or NOX inhibition can ameliorate mutant ATXN7 toxicity.

## Background

Spinocerebellar ataxia type 7 (SCA7) is an autosomal dominant inherited neurodegenerative disorder characterized by cerebellar ataxia and visual problems due to a progressive loss of neurons within the cerebellum, retina and brainstem [1,2]. Expansion of an unstable CAG repeat in the first coding exon of the SCA7 gene, resulting in an expanded polyglutamine domain in the N-terminal of the ataxin-7 (ATXN7) protein causes the disease [3]. The ATXN7 protein is widely expressed in the nervous system [4-6] and is a subunit of the STAGA (SPT3-TAF(II)31-GCN5L acetylase) complex [7,8].

To date, nine disorders including Huntington’s disease (HD), dentatorubral and pallidoluysian atrophy (DRPLA), spinobulbar muscular atrophy (SBMA) and six forms of Spinocerebellar ataxias (SCA1-3, 6, 7 and 17) caused by expanded polyglutamine domains have been identified, for review see [9]. These disorders are commonly known as polyglutamine (polyQ) diseases and are characterized by the aggregation of the expanded polyQ protein. Mutant ATXN7 has been shown to aggregate and form inclusions in both patients and cell models [5,6,10]. A correlation between the ability of polyglutamine proteins to aggregate and toxicity has been shown, however, whether misfolded monomers, oligomers or large inclusions formed during the aggregation process are the major toxic species is still unclear [11-13].

Oxidative stress arise when the levels of free radicals exceed the capacity of the cell’s endogenous anti-oxidant systems and result in damage to cellular components like DNA, lipids and proteins [14]. Prolonged oxidative stress can lead to cell death and this pathway has been implicated in many neurodegenerative diseases including Alzheimer’s disease (AD), Parkinson’s disease (PD) and amyotrophic lateral sclerosis (ALS), for review see [15,16]. Oxidative stress has also been implicated in polyglutamine diseases, for instance Huntington’s disease, DRPLA and SCA1 [17-20]. ROS (reactive oxygen species) is a major type of free radicals in cells and is normally produced during oxidative phosphorylation in mitochondria [14]. Up to 2% of the electrons passing the electron transport chain escape and reacts with molecular oxygen to yield ROS. Due to their high metabolic rate neurons are exposed to high levels of ROS [14]. Other sources of ROS is NADPH oxidase (NOX) complexes and enzymes like xanthine oxidase, lipooxygenase and cyclooxygenase [21]. NOX complexes produce superoxide anions and are present in neurons as well as astrocytes and microglia [22]. To prevent damage from excess ROS, neurons and other cells have an extensive anti-oxidant defense system made up of several enzymes and small molecules [23]. Enzymes with anti-oxidant function include glutathione transferases (GSTs), super oxide dismutases (SODs), superoxide reductase, catalase (CAT), peroxiredoxins (Prxs) and glutathione peroxidases (Gpxs). Glutathione (GSH) an important small anti-oxidant molecule protects cells against oxidative stress by conjugating with ROS in a reaction catalyzed by GST [24]. During the conjugation reaction GSH become oxidized into glutathione disulfide (GSSH) and is thus depleted. GSH depletion have been shown to occur in neurodegenerative disorders like Parkinson’s disease [25]. In mammals, there are at least three forms of SOD: a cytosolic (CuZnSOD/SOD1), a mitochondrial (MnSOD/SOD2) and an extracellular (ECSOD/SOD3) form. SOD enzymes catalyze the dismutation of superoxide, a primary ROS, into hydrogen peroxide [26]. The hydrogen peroxide is then further converted to water and oxygen by CAT, Prxs or Gpxs [14,27].

In this study, we show that oxidative stress plays a major role in ATXN7-induced toxicity using a new stable inducible PC12 cell model. We found that induction of mutant ATXN7Q65-GFP expression led to a concomitant increase in ROS levels and aggregation of the disease protein followed by decreased cell viability a few days later. Analysis of some key anti-oxidant defense enzymes revealed decreased levels of catalase, which could contribute to decreased clearance of ROS. Furthermore, inhibition of NOX complexes prevented the increase in ROS and ameliorated aggregation suggesting that mutant ATXN7 increase the ROS levels by activating this complex. Moreover, supporting the cells through application of exogenous anti-oxidants or inhibition of NOX complexes ameliorated AXTN7Q65 induced toxicity.

## Results

### Expression of mutant ATXN7 leads to oxidative stress followed by toxicity in an inducible SCA7 PC12 cell model

**Figure 1:**
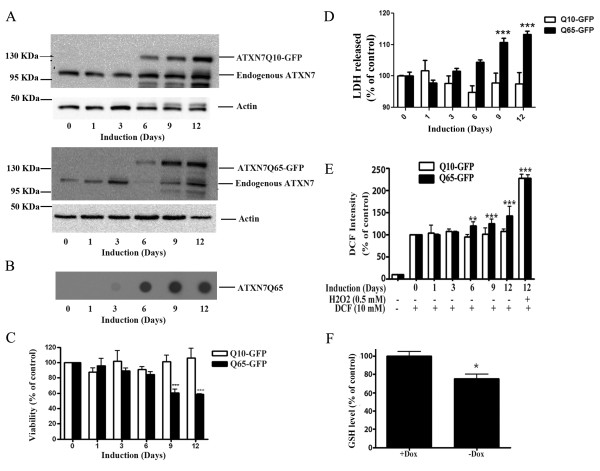
**Expression of ATXN7Q65-GFP leads to increased ROS levels prior to toxicity. A**) Western blot analysis of ATXN7 in FLQ10 and FLQ65 stable PC12 cell lines induced to express GFP-tagged ATXN7 with 10 (ATXN7Q10-GFP, top panel) or 65 (ATXN7Q65-GFP, lower panel) glutamines for the indicated number of days. Actin was used as loading control. **B**) Analysis of ATXN7 aggregation in FLQ65 cells induced to express ATXN7Q65-GFP for 1–12 days using the filter trap assay. **C**) Viability measured by the WST-1 assay and normalized against the protein content in FLQ10 and FLQ65 cells induced to express ATXN7Q10-GFP or ATXN7Q65-GFP for the indicated number of days. **D**) Toxicity measured by the LDH membrane leakage assay in FLQ10 and FLQ65 cells induced to express ATXN7Q10-GFP or ATXN7Q65-GFP for the indicated number of days. **E**) Total cellular ROS levels measured using DCHF-DA in cells induced to express ATXN7Q10-GFP or ATXN7Q65-GFP for 0–12 days. **F**) Analysis of GSH levels in cells induced (−Dox) to express ATXN7Q65-GFP for 9 days and non-induced control cells (+Dox). For quantifications all data are shown as means ± SEM from three independent experiments with triplicates. * p <0.05, ** p <0.01 and *** p <0.001.

To study the impact of mutant ATXN7 on cellular functions we used two recently generated stable inducible PC12 cell lines expressing N-terminal FLAG- and C-terminal GFP-tagged ATXN7 with 10 (FLQ10 line) or 65 (FLQ65 line) glutamines [28]. In these cell lines the expression of the corresponding transgenic proteins named ATXN7Q10-GFP and ATXN7Q65-GFP is controlled by the Tet-off expression system and induced by removal of doxycycline from the cell culture media. The induction timing, expression levels and sub-cellular localization of the transgenic ATXN7-GFP proteins have previously been extensively characterized and showed not to differ in these two cell lines [28]. Immunoblotting with an ATXN7 antibody revealed weak expression after three days of induction, but clear expression of both constructs from day 6 onwards (Figure 1A and [28]. No ATXN7 aggregation was detected in ATXN7Q10-GFP expressing cells [28]. However, in cells expressing ATXN7Q65-GFP filter trap analysis revealed aggregation from day 3 onwards and from day 9 the level of aggregated ATXN7 material was stable (Figure 1B and [28]).

Analysis of cell viability/toxicity revealed a progressive decrease in viability and increased toxicity as expression of ATXN7Q65-GFP was induced, see Figure 1C-D. A significant decrease in viability was observed from day 9 after induction using the WST-1 viability assay (Figure 1C). In accordance with this a statistical increase in toxicity, measured as membrane leakage, could also be observed on day 9 after induction (Figure 1D). In contrast, expression of the ATXNQ10-GFP protein did not result in any significant change in cell viability or toxicity on any day (Figure 1C-D). To establish whether induction of oxidative stress by ATXN7Q65-GFP could play a role in the decreased cell viability, we measured total cellular ROS levels at various time points after induction of ATXN7Q65-GFP expression. We found that the ROS levels increased in a time dependent manner after induction of ATXN7Q65-GFP expression (Figure 1E). A significant increase in ROS was first observed at day 6, prior to the decrease in viability of the ATXN7Q65-GFP expressing cells. In contrast, expression of ATXNQ10-GFP protein did not result in any significant change in total cellular ROS levels (Figure 1E). As a positive control increased levels of ROS could be observed in both FLQ10 and FLQ65 cells after treatment with hydrogen peroxide (Figure 1E).

To further confirm that expression of ATXN7Q65-GFP resulted in an oxidative environment, we measured the GSH level in FLQ65 cells induced to express ATXN7Q65-GFP for nine days. A significant reduction in the GSH level was observed (Figure 1F). Taken together, these observations suggest that expression of ATXN7Q65-GFP results in increased ROS levels and oxidative stress prior to any observable toxicity.

### Anti-oxidant treatment rescues ATXN7Q65-GFP toxicity

**Figure 2:**
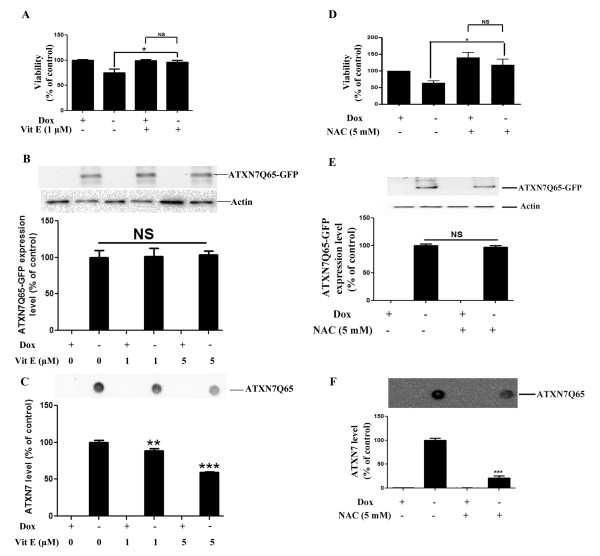
**Antioxidant treatment rescues cells from ATXN7Q65-GFP induced toxicity and decrease ATXN7 aggregation. A)** Viability measured by WST-1 in FLQ65 cells not induced (+Dox) or induced (−Dox) to express ATXN7Q65-GFP for 9 days while growing in media with or without 1 μM Vitamin E (α-tocopherol). **B**) Analysis and quantification of soluble ATXN7Q65-GFP by western blot in FLQ65 cells grown and treated as in A. **C**) Analysis and quantification of ATXN7 aggregation by filter trap assay in FLQ65 cells grown and treated as in A. **D**) Viability in cells grown as in A but treated with or without 5 mM NAC. **E**) Analysis and quantification of soluble ATXN7Q65-GFP by western blot in FLQ65 cells grown and treated as in D. **F**) Analysis and quantification of ATXN7 aggregation by filter trap assay in FLQ65 cells grown and treated as in D. For all western blots actin was used as loading control. For quantifications data are shown as means ± SEM from three independent experiments with triplicates. NS: not significant, * p <0.05, ** p <0.01 and *** p <0.001.

To confirm that the oxidative stress conditions induced by ATXN7Q65-GFP played a role in the decreased cell viability, FLQ65 cells induced to express ATXN7Q65-GFP for nine days while growing in media supplemented or not supplemented with the anti-oxidants α-tocopherol (Vitamin E) or NAC were analyzed. Treatment with either anti-oxidant rescued the viability of ATXN7Q65-GFP expressing cells and there was no longer any difference in viability between non-induced and induced FLQ65 cells expressing ATXN7Q65-GFP (Figure 2A and 2D). The positive effects of the anti-oxidant treatments were not due to changes in ATXN7 expression, as neither treatment did alter the expression level of soluble ATXN7Q65-GFP (Figure 2B and 2E). This result suggests that oxidative stress is contributing to the toxicity in SCA7 and this effect can be counteracted by application of an anti-oxidant.

### Oxidative stress affects aggregation of mutant ATXN7

**Figure 3:**
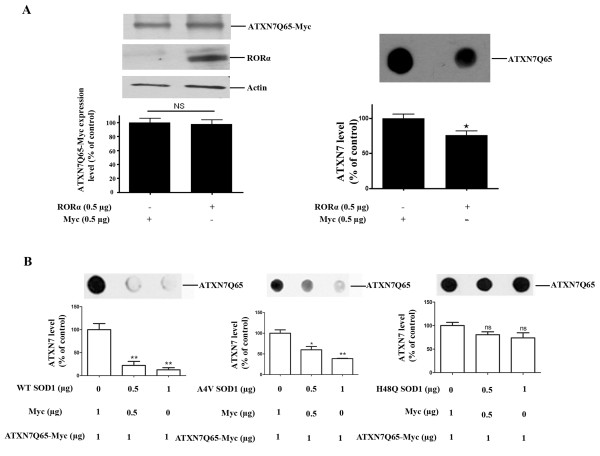
**RORα and SOD1 reduce ATXN7Q65-Myc protein aggregation. A**) HEK 293 cells were co-transfected to express ATXN7Q65-Myc and RORα. After 48 hours RORα and ATXN7Q65-Myc expression (left panel) and ATXN7 aggregation (right panel) were analyzed and quantified. Actin was used as loading control for western blots. **B**) HEK 293 T cells were co-transfected with ATXN7Q65-Myc and 0–1 μg of a plasmid encoding wild-type (WT), A4V mutant or H48Q mutant SOD1. Empty vector (Myc) was used to allow the same amount of plasmids to be transfected in each well. Forty-eight hours after transfection ATXN7Q65-Myc aggregation was analyzed by filter trap in cells co-transfected with WT SOD1 (left), A4V SOD1 (middle) and H48Q SOD1 (right). All quantifications are shown as means ± SEM from three independent experiments with triplicates. NS: not significant, * p <0.05 and ** p <0.01.

**Figure 4:**
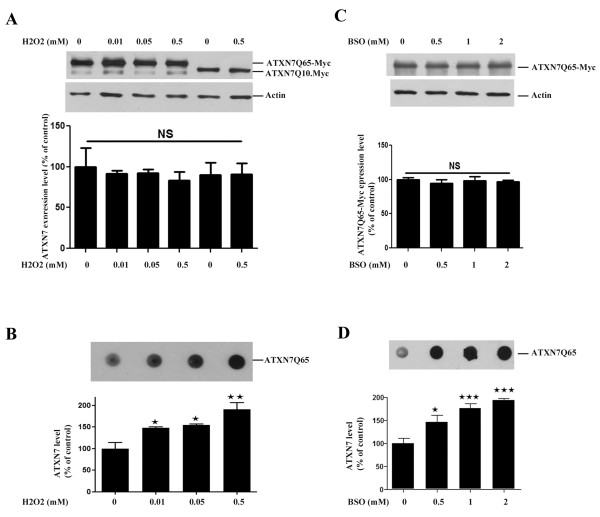
**Oxidative stress increases aggregation of ATXN7Q65-Myc. A**) HEK 293 cells transfected to express ATXN7Q10-myc or ATXN7Q65-myc was treated with 0–0.5 mM H_2_O_2_ for 48 h and ATXN7 levels were analyzed by western blot. Upper panel; a representative western blot, lower panel: quantification of ATXN7 expression levels. **B**) Analysis of aggregated ATXN7 in cells transfected and treated as in A. Upper panel; a representative blot, lower panel; quantification of ATXN7 aggregation. **C**) HEK 293 cells transfected as in A was treated with 0–2 mM BSO for 48 h and western blot performed. Upper panel; a representative western blot, lower panel; quantification of ATXN7 expression levels. **D**) Analysis of aggregated ATXN7 in cells treated as in C. Upper panel; a representative blot, lower panel; quantification of ATXN7 aggregation. Actin was used as loading control in all western blot experiments. All quantitative data are shown as means ± SEM from three independent experiments with triplicates. NS: not significant, * p <0.05, ** p <0.01 and *** p <0.001.

To investigate the relationship between oxidative stress and ATXN7 aggregation, we analyzed whether anti-oxidant treatment of our stable FLQ65 cells induced to express ATXN7Q65-GFP also had an effect on the level of ATXN7 aggregation. Results showed that both α-tocopherol and NAC treatment during the induction of ATXN7Q65-GFP expression in the stable PC12 cell model lowered the level of aggregated ATXN7 material with circa 40–80% (Figure 2C and 2F). To further confirm the connection between oxidative stress and ATXN7 aggregation and make sure that the effect seen in Figure 2 was not influenced by the GFP-tag on ATXN7 or specific to PC12 cells, we did further experiments in HEK 293 T cells transfected to express myc-tagged ATXN7 with 10 (ATXN7Q10-myc) or 65 (ATXN7Q65-myc) glutamines. We first investigated whether support of the anti-oxidant system could also ameliorate the aggregation of ATXN7Q65-myc, by co-transfecting the HEK 293 T cells with ATXN7Q65-myc and RORα or SOD1. Over-expression of RORα, a transcription factor known to activate anti-oxidant genes [29], ameliorated the aggregation of ATXN7Q65-Myc (Figure 3A). So did over-expression of wild-type SOD1 with full dismutase activity, whereas co-expression of mutant forms of SOD1 with reduced (A4V) [30,31] or no enzymatic activity (H48Q) [31] showed reduced or no ability, respectively, to reduce ATXN7Q65-Myc aggregation (Figure 3B). Neither RORα nor SOD1 co-expression affected the expression of soluble ATXN7Q65-Myc (Figure 3A and data not shown). We next investigated whether promotion of an oxidative environment could aggravate the aggregation of ATXN7Q65-myc by treating transfected HEK 293 T cells with increasing concentrations of H_2_O_2_ (Figure 4A-B) or BSO (Figure 4C-D) an inhibitor of GSH biosynthesis [32]. No trace of aggregated material could be detected in ATXN7Q10-Myc expressing cells under control or treated conditions (data not shown). However, aggregated ATXN7 material was detected in ATXN7Q65-Myc cells and both H_2_O_2_ and BSO treatment led to an increase in aggregated material without affecting the expression of soluble ATXN7Q65-Myc (Figure 4). Taken together, these data suggest that there is a clear connection between oxidative stress and aggregation of mutant ATXN7.

### Expression of ATXN7Q65-GFP results in changed expression levels of some key anti-oxidant enzymes

**Figure 5:**
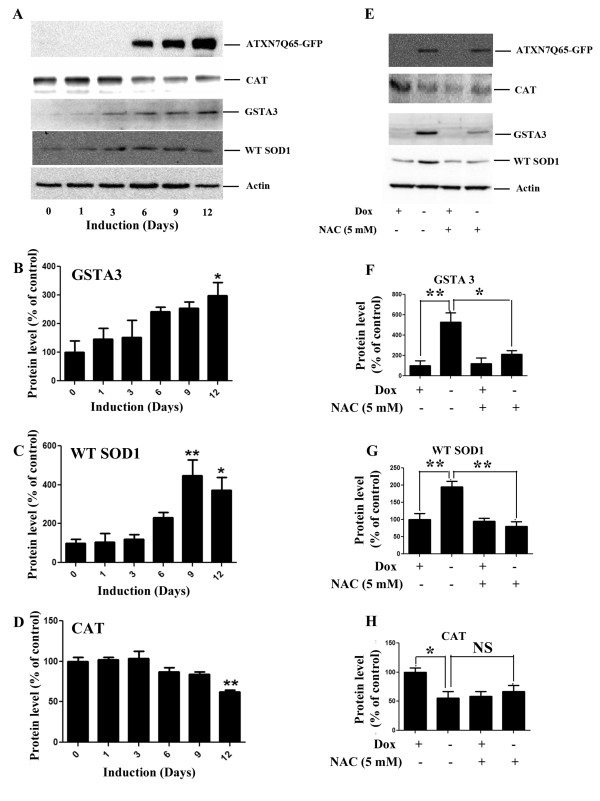
**Reduced CAT levels, but increased levels of SOD1 and GSTA3 in ATXN7Q65-GFP expressing cells. A**) Representative western blot analysis of GSTA3, SOD1 and CAT levels in FLQ65 cells induced to express ATXN7Q65-GFP for 0–12 days. **B**) Quantitative analysis of GSTA3 levels from three experiments as shown in A. **C**) Quantitative analysis of SOD1 levels from three experiments as shown in A. **D**) Quantitative analysis of CAT levels from three experiments as shown in A. **E**) NAC effect on the expression of GSTA3, SOD1 and CAT levels in ATXN7Q65-GFP expressing cells. FLQ65 cells not induced or induced to express ATXN7Q65-GFP for 9 days while growing in media with or without NAC (5 mM) were analyzed. **F**) Quantitative analysis of GSTA3 levels from three experiments with treatments as in E. **G**) Quantitative analysis of SOD1 levels from three experiments with treatments as in E. **H**) Quantitative analysis of CAT levels from three experiments with treatments as in E. All quantifications are shown as means ± SEM from three independent experiments with triplicates. * p <0.05, ** p <0.01 and *** p <0.001.

Mutant ATXN7 could induce oxidative stress by interfering with the anti-oxidant defense system or by causing an increase in free radical production. To investigate the status of the anti-oxidant defense system, we analyzed the expression levels of some key anti-oxidant enzymes; Glutathione transferase A3 (GSTA3), SOD1 and CAT in our stable PC12 model. After induction of ATXN7Q65-GFP, the expression levels of GSTA3 and SOD1 showed a progressively increasing trend with statistical differences in expression at day 9 and/or 12 after induction (Figure 5A-C). In contrast, the expression level of CAT showed a decreasing trend after induction of ATXN7Q65-GFP (Figure 5A and 5D). We also investigated whether anti-oxidant treatment could prevent the change in expression of these enzymes. Indeed, in induced FLQ65 cells grown in NAC supplemented media for 9 days, the increase in GSTA3 and SOD1 expression was reduced or completely gone (Figure 5E-G). However, the decreased expression of CAT was not restored (Figure 5H). Our results suggest that the cell is trying to cope with the oxidative environment by up-regulating at least some key anti-oxidant enzymes.

### Increased ROS production from NOX complexes in ATXN7Q65-GFP expressing cells

**Figure 6:**
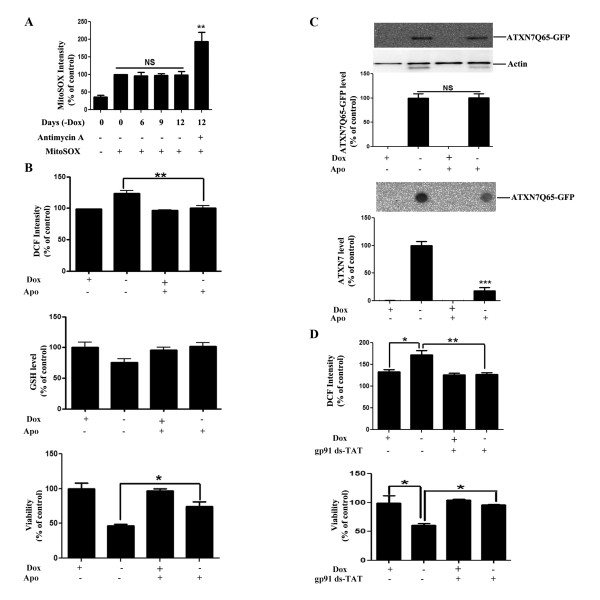
**Inhibition of NOX complexes reduces ROS production, increase the GSH level and ameliorates toxicity. A**) Measurement of mitochondrial superoxide levels using MitoSox in cells induced to express ATXN7Q65-GFP for 0, 6, 9 or 12 days. Antimycin A treatment was used as a positive control. **B**) Effect of NOX complex inhibition on ATXN7Q65-GFP cells. ROS levels (top panel), GSH levels (middle panel) and cell viability (lower panel) was analyzed in FLQ65 cells not induced (+Dox) or induced (−Dox) to express ATXN7Q65-GFP for 9 days while growing in media with or without the NOX complex inhibitor apocynin (50 μM). **C**) Effect of NOX inhibition by apocynin on ATXN7 aggregation and ATXN7Q65-GFP expression in FLQ65 cells grown and treated as in A. Top panel; representative western blot and quantification of expression. Lower panel; representative dot blot and quantification of aggregation. **D**) Effect of NOX complex inhibition by gp91ds-TAT on ATXN7Q65-GFP cells. ROS levels (top panel) and cell viability (lower panel) was analyzed in FLQ65 cells not induced (+Dox) or induced (−Dox) to express ATXN7Q65-GFP for 9 days while growing in media with or without 10 μM of the inhibitor peptide. All quantifications are shown as means ± SEM from three independent experiments with triplicates. NS: not significant, * p <0.05, ** p <0.01 and *** p <0.001.

Mitochondria are a major source of ROS and damage to mitochondria resulting in increased mitochondrial superoxide anion production has been implicated in neurodegeneration. To investigate whether this mechanism plays a role in the elevation of the ROS levels in our ATXN7Q65-GFP expressing cells, we measure the level of mitochondrial superoxide anions at various time points after induction of ATXN7Q65-GFP expression. No increase in mitochondrial superoxide production could be observed at any time point after ATXN7Q65-GFP induction (Figure 6A). Antimycin A treatment was used as a positive control and elevated the levels of mitochondrial ROS as expected (Figure 6A).

We then went on to investigate whether an increased ROS production by NOX complexes contributes to the oxidative stress environment induced by ATXN7Q65-GFP. The level of ROS, GSH and ATXN7 aggregation, as well as viability, was measured in FLQ65 cells induced to express ATXN7Q65-GFP for nine days while growing in media supplemented or not supplemented with a NOX complex inhibitor apocynin (Apo). The apocynin treatment completely blocked the increase in ROS, led to a small increase in the level of GSH, reduced ATXN7 aggregation and ameliorated the toxicity in ATXN7Q65-GFP expressing cells (Figure 6B-C). Similarly treatment of the ATXN7Q65-GFP expressing cells with the cell-permeable gp91ds-TAT peptide, a specific NOX complex inhibitor [33], also decreased ROS levels and ameliorated the toxicity (Figure 6D). In contrast, treatment with the control peptide Scramble-TAT had no effect on ROS levels or viability (data not shown). These results suggest that mutant ATXN7 either directly or indirectly leads to activation of NOX complexes resulting in increased ROS production and oxidative stress in our cell model.

## Discussion

Oxidative stress has been implicated in the pathology of several neurodegenerative diseases. In this study we show that oxidative stress is a major contributor to aggregation and toxicity of the SCA7 disease protein ATXN7. Furthermore, our results suggest that mutant ATXN7 could cause oxidative stress by directly or indirectly reducing the levels of the anti-oxidant enzyme CAT and increasing the ROS production through NOX complexes. Most importantly we show that anti-oxidant treatment or inhibition of ROS production from NOX complexes ameliorates ATXN7 toxicity.

To study the role of oxidative stress in SCA7, we used a new stable inducible cell model (FLQ65) in which the expression of GFP-tagged ATXN7 with an expanded glutamine domain (ATXN7Q65-GFP) is controlled by the Tet-off system. In this model, clear expression and aggregation of ATXN7Q65-GFP was detected six days after induction (doxycycline removal from the media), ROS levels was increased from day 6 and the cell viability decreased from day 9 onwards. The increase in ROS levels hence preceded cell toxicity in our model and anti-oxidant treatment not only reduced ROS levels but also completely rescued the viability of our cells. This suggests that mutant ATXN7 cause toxicity by inducing oxidative stress. Other polyglutamine proteins have also been shown to cause elevated ROS levels and oxidative damage to DNA and lipids have been reported in both HD patients and animal models, for review see [34]. However, the mechanism(s) by which expanded polyglutamine proteins alter the redox-homeostasis is unclear. Increased ROS production either due to aggregating structures functioning as centers for oxidative reactions [35,36] or polyglutamine proteins causing damage to the mitochondria electron transport chain [37,38] has been suggested. In our study we saw no evidence for increased ROS production from mitochondria. However, we found that inhibition of NOX complexes prevented the ATXN7Q65-GFP induced elevation of ROS and ameliorated the ATXN7 toxicity. This is in agreement with a recent study by Bertoni et al. [39], suggesting that expression of an expanded polyQ stretch can lead to NOX activation. NOX complexes have been mostly studied in immune cells where their production of large burst of superoxide participates in the killing of invading microorganisms. However, NOX complexes are also present in neurons and PC12 cells, and have been suggested to regulate neurite outgrowth and neuronal activity [22,40]. SOD1 is a key enzyme for clearing away superoxide anions and convert the radical into molecular oxygen and H_2_O_2_[41]. Following this dismutase reaction, the H_2_O_2_, which is also reactive, is cleared away by catalase or other enzymes like glutathione peroxidases [42]. In our study we observed an increased expression of SOD1, which is most likely an adaptive response to increased superoxide levels. Similarly we could observe increased level of GSTA3, an enzymes that detoxify oxidatively damaged molecules for instance lipid peroxidation products [24]. However, we could also observe a decrease in the expression of catalase which could compromise the clearance of H_2_O_2_ and contribute to the generation of an oxidative environment. In agreement with our finding, decreased catalase activity have been reported in fibroblast cultures of HD patients [43] and decreased catalase expression was also observed in a HD cell model by Reijonen et al [20]. However, in the Reijonen study they also in contradiction to our results observed a decrease in several other anti-oxidant enzymes including SOD1 and SOD2 [20]. The contradictory results between our study and Reijonen et al could reflect differences between mutant huntingtin and ATXN7 or differences in expression length and levels in our stable model and their transient transfection model. In fact, increased levels of several antioxidants including SOD2, peroxiredoxins and glutathione peroxidases have also been reported in HD patients by Sorolla et al [44]. However, comparing our results with the data from the study by Sorolla et al also shows differences, as Sorolla et al identified increased levels of catalase in HD brain [44]. Again the difference between the Sorolla study and our data could reflect differences between huntingtin and ATXN7. It is possible that mutant ATXN7 as a subunit in the co-activator complex STAGA might specifically effect the regulation of catalase gene expression. Interestingly, we could see that the decrease in catalase levels in our mutant ATXN7 cells could not be reversed by anti-oxidant treatment even though this prevented the increase in ROS and reversed the changes in SOD1 and GSTA3 levels. This suggests that the mechanism by which mutant ATXN7 effect catalase levels could be different than the mechanism(s) altering SOD1 and GSTA3. Taken together, it seems clear that mutant ATXN7 as many other polyglutamine proteins induce oxidative stress and changes in the anti-oxidant defense system. However, which components of the anti-oxidant system are altered and how these alterations contributes to reduce or worsen the oxidative stress and toxicity induced by the different polyglutamine proteins is still more unclear and requires more investigation.

Providing anti-oxidant support have been suggested as a potential therapeutic approach for polyglutamine disease, for review see [34]. Consistent with this idea treatment with a general anti-oxidant or counteracting the increased ROS production by inhibition of NOX complexes not only ameliorated the toxicity of mutant ATXN7, but also reduced the level of aggregated ATXN7 in our SCA7 model. In contrast, treatment with oxidative stress inducers (H_2_O_2_ or BSO) elevated ATXN7 aggregation. Hence there is a strong correlation between oxidative stress and ATXN7 aggregation. Misfolding and aggregation of polyQ-expanded protein is believed to be a key step in the pathogenesis of polyQ-diseases [45,46]. However, whether misfolded monomers, oligomers or large inclusions formed during the aggregation process are the major toxic species is still unclear [11-13]. Furthermore, proteolytic cleavage of several polyglutamine proteins generating more aggregate prone and toxic fragments has been reported [45,46]. In SCA7, cleavage of ATXN7 by caspase-7 at amino acids 266 and 344 has been reported [47]. In our model we could observe N-terminal ATXN7 fragments [28] and these, as well as the full-length protein and aggregated material is present at the time when we observe increase in ROS levels. Furthermore, we have seen that full-length ATXN7 is predominately localized to the nucleus, whereas these N-terminal fragments show a more cytoplasmic localization [28]. Since NOX complexes are mostly localized in cytoplasmic vesicles or in the plasma membrane [22], it is tempting to speculate that if ATXN7 activates NOX complexes through a direct interaction, then it is possibly the N-terminal mutant ATXN7 fragments which do so either in a soluble or aggregated form. This would be in line with the observation by Young et al. showing higher cellular toxicity by N-terminal ATXN7 fragments [47]. Clearly future studies are needed to determine the exact mechanism and which form of mutant ATXN7 that induces the NOX activity.

## Conclusions

Taken together, our work suggests that mutant ATXN7 induce oxidative stress by reducing the level of the anti-oxidant enzyme CAT and increasing the ROS production from NOX complexes. We show that there is a clear correlation between ROS levels, mutant ATXN7 aggregation and decreased viability. Moreover, inhibition of NOX complexes or treatment with an anti-oxidant can ameliorate the mutant ATXN7 toxicity.

## Methods

### Plasmids

Plasmids FLQ10 and FLQ65, encoding N-terminal Flag and C-terminal myc tagged full-length ataxin-7 referred to as ATXN7Q10-Myc and ATXN7Q65-Myc have been previously reported [10]. Plasmids encoding GFP-tagged WT, A4V or H48Q SOD1 constructs as well as RORalpha have been previously described [10,48].

### Cell culture and transfections

Generation of stable inducible PC12 cell lines expressing N-terminal FLAG- and C-terminal GFP-tagged ATXN7 with 10 (FLQ10 line) or 65 (FLQ65 line) glutamines have been described previously [28]. In these cell lines expression of the corresponding proteins named ATXN7Q10-GFP and ATXN7Q65-GFP is induced upon removal of doxycline from the media. The FLQ10 and FLQ65 stable PC12 cell lines were grown at 37°C and 5% CO2, in DMEM (Invitrogen) supplemented with 10% horse serum (Invitrogen), 5% Tet System Approved fetal bovine serum (PAA), 100 μg/ml G418 (Invitrogen), 100 units/ml penicillin G sodium, 100 μg/ml streptomycin sulphate (Invitrogen), 100 μg/ml hygromycin (Invitrogen) and 1 μg/ml doxycycline (Sigma) when desired.

Human Embryonic Kidney 293 T (HEK 293 T) cells were maintained in Dulbecco’s modified Eagle’s medium (DMEM, Invitrogen) supplemented with 10% fetal bovine serum (FBS, Invitrogen) and 1% penicillin/streptomycin (PEST, Invitrogen) at 37°C, 5% CO2.

For transient transfections, 7 × 10^5^ HEK 293 T cells were seeded in 6 well plates and transfected 24 h later using Polyethylenimine (CellnTec) according to the product protocol.

### Treatments and synthesis of gp91-TAT

Cells were treated with various concentration of H_2_O_2_ (0–0.5 mM) (Sigma), NAC (N-acetyl-L-cysteine) (0–5 mM) (Sigma) or BSO (buthionine sulfoximine) (0–2 mM) (Sigma). Apocynin, and α-tocopherol were used at a final concentration of 50 μM and 1 μM, respectively. The NOX complex inhibitor peptide gp91ds-TAT ([H]-R-K-K-R-R-Q-R-R-R-C-S-T-R-I-R-R-Q-L-NH_2_) and the control peptide Scramble-TAT ([H]-R-K-K-R-R-Q-R-R-R-C-L-R-I-T-R-Q-S-R-NH_2_) has been previously described [33] and were used at a concentration of 10 μM. The peptides were synthesized (SYRO multiple peptide synthesizer, MultiSynTech, Germany) on Fmoc-Rink- amide-chemmatrix resin (PCAS biomatrix inc.) using standard Fmoc solid-phase peptide synthesis. The peptide was cleaved using 95% TFA/2% water/2% triisopropylsilane/1% 1,2-ethanedithiol) for 3 h and precipitated in diethylether. The crude peptide was dried in vacuum overnight. The peptide was purified by HPLC on a Discovery® C-18 Supelco® column (Sigma-Aldrich, Sweden) using a gradient of acetonitrile/ water containing 0.1% TFA. Purity and identity was verified by analytical HPLC and by MALDI-TOF on a Voyager STR. The mass-spectrum was acquired in positive ion reflector mode using a-cyano-4-hydroxycinnamic acid as matrix (Sigma-Aldrich) (10 mg/ml, 7:3 acetonitrile: water, 0.1% TFA).

### Cell lysis and Western blotting

Cell lysis and Western blotting was done as previously described [28]. In brief, cells were lysed with RIPA buffer (Millipore) supplemented with protease inhibitors and the supernatant collected after centrifugation at 21,000 g at 4 °C for 10 min. Protein concentrations were determined with Bradford assay (Bio-Rad) and 10–20 μg of extract was subjected to SDS–PAGE. Proteins were transferred onto nitrocellulose membrane (Whatman), the membrane blocked and incubated with primary antibodies in 2% milk-TBST (100 mM Tris-buffered saline pH 7.4, 0.1% tween-20). Membranes were then washed, incubated with secondary antibody in 2% milk-TBST, and again washed with TBST. The protein of interest was visualized using SuperSignal West Pico chemiluminescent substrate or SuperSignal West extended duration substrate kits (Pierce) followed by film exposure or detection by a ChemiDoc XRS + imaging system (BioRad). Primary antibodies were used at the following concentrations; Ataxin-7 [10] 1:700, actin 1:500 (SC-1616, Santa Cruz), CAT 1:500 (SC-50508, Santa Cruz), SOD1 1:500 (SC-11407, Santa Cruz) and GSTA3 1:500 (gift from B. Mannervik). Signal intensities of target bands were quantified by Image lab software (BioRad). The relative intensity of the target protein in control and treated samples were acquired by first normalizing the target band with the corresponding actin intensity. The normalized intensity in control or treated samples was then divided by the sum of the normalized intensities of the target protein in control and all treated samples. The quote for the control sample was set to 100% and all treated samples in that experiment is shown as percent compared to control.

### Filter trap assay

Filter trap assay was done as previously described [28]. In short, cells were lysed in RIPA buffer and the pellets obtained after centrifugation at 21,000 g for 10 min were washed and resuspended in 50 μl DNAseI reaction buffer containing four unit of DNaseI enzyme (EN0521, Fermentas). The resuspended pellet, called the insoluble fraction, was incubated at 37 °C for 1 hr and Bradford assay (Bio-Rad) then used to determine the protein concentration in the sample. SDS and DTT were then added to a final concentration of 2% and 100 mM respectively before samples were heated at 95 °C for 5 min. Insoluble fractions were loaded and vacuum filtered through a 0.2 μm pore size membrane using a Bio-Rad dot-blot apparatus and a 0.1% SDS solution was added to each dot-blot slots twice to wash. The membrane was then removed from the dot-blot, blocked and subjected to immunoblotting using ATXN7 antibody as described above. Following immunoblotting, signal intensities of ATXN7 dots were quantified by Image lab software (BioRad) and normalized against the protein concentrations. For quantification of aggregation levels the average intensity from each dot was divided by the sum of the intensities of ATXN7 from all time points in that experiment and the intensity from the untreated control sample was set to 100%. All treated samples in that experiment is shown as percent compared to control.

### Measuring of total ROS levels

FLQ10 or FLQ65 cells grown without Dox for 0, 2, 5, 8 or 11 days were seeded into 96-well culture plates in triplicate and grown in –Dox media for another 24 h before ROS was measured. Cells grown with doxycyline were used as control. ROS levels were measured by the oxidation-sensitive probe, dichloro-fluorescein-diacetate (DCHF-DA) (Sigma). Briefly, cells were washed two times with PBS after which PBS containing DCFH-DA (10 mM) was added to three wells with cells, whereas PBS only was added to three control wells. Fluorimetric measurement was taken immediately after addition for a period of 30 min using a flex station II plate reader. Cells treated with H_2_O_2_ (0.5 mM) were used as a positive control. The ROS levels were determined by subtracting the fluorescence of the PBS only treated well from the fluorescence of the DCFH-DA treated well to remove any potential background from GFP fluorescence.

### Detection of mitochondria superoxide levels

FLQ65 cells grown without Dox for 5, 8 or 11 days were seeded into 96-well culture plates in triplicates and grown in –Dox media for another 24 h before mitochondria superoxide was measured. Cells grown with dox were used as control. Mitochondria superoxide levels were measured by the oxidative sensitive probe, MitoSOX™ Red reagent (Sigma). Briefly, cells were washed two times with PBS after which PBS containing MitoSOX™ Red reagent (5 μM) was added. Cells were incubated for 30 min after which fluoremetric measurement was taken for a period of 30 min using a flex station II plate reader. Cells treated with Antimycin A (100 nM) were used as a positive control.

### Cell viability/toxicity measurements

WST-1 viability assays (Clontech) were performed according to the manufacturer’s protocol. Briefly, 50,000 induced or non-induced, treated or non-treated FLQ10 or FLQ65 PC12 cells were seeded in 96-well cell culture plate 24 h before viability measurements. Ten μl of WST-1 reagent was added to each well and after two hours of incubation the absorbance (450–690 nm) was measured on a Digiscan absorbance reader (Labvision). Following the WST-1 assay the protein concentration in each well was determined using Lowry assay (Bio-Rad). The obtained cell viabilities were normalized by protein concentration, and the value obtained from untreated non-induced sample was set to 100%.

Membrane integrity as a measure of toxicity was determined by analysis of lactate dehydrogenase (LDH) leakage from the cytosol of damaged cells using the CytoTox-ONE™ homogeneous membrane integrity assay (Promega), according to the manufacturer’s protocol. Briefly, 50,000 induced or non-induced cells were seeded in 12-well cell culture plate 24 h before analysis. Fifty μl of media from each sample/well was transferred to 96 well plates and incubated at room temperature for 20 min. A hundred μl of CytoTox-ONE (Promega) reagent was added to each sample and fluorescence measured using a flex station II plate reader at excitation wavelength of 560 nm and emission wavelength of 590 nm.

### GSH assay

GSH level was measured using the GSH kit (Promega). Briefly, FLQ65 cells grown with or without Dox and treated with 50 μM Apo for 9 days were washed 2 times with PBS and lysed with GSH-Glo™ Reaction Buffer (Promega) for 30 min. Lysates were diluted 1:15 in deionized water and 10 μl of diluted lysate was transferred to 96-well plate in duplicates. Hundred μl of 1X GSH-Glo™ Reagent was added to each well and the samples incubate for 30 min at room temperature before 100 μl of prepared Luciferin Detection Reagent (Promega) was added to each well. After a 15 min incubation the luminescence was read using a microplate luminometer (Promega). The obtained luminescence value was normalized by protein concentration, and the value obtained from untreated non-induced sample was set to 100%.

### Statistical analysis

Statistical analysis was done by one-way ANOVA followed by Tukey’s post-hoc test using Prism graph pad 5.0 or by two-tailed student *t* test. Data is represented as mean ± standard error of at least three independent experiments. In all cases, P <0.05 was considered to be statistically significant. Data are expressed as a percentage of control unless otherwise stated.

## Abbreviations

ATXN7, Ataxin-7; CAT, Catalase; GSH, Glutathione; GST, Glutathione transferase; NOX, NADPH oxidase; ROS, Reactive oxygen species; SCA7, Spinocerebellar ataxia type 7; SOD, Super oxide dismutase.

## Competing interests

The authors have no conflict of interest to declare.

## Authors’ contributions

AA carried out experiments, participated in design of the study and drafted the manuscript. XY carried out western blot and filter trap assays on HEK293T cells and participated in the design of the study. SL synthesized peptides and aided in experimental design. ÜL participated in experimental design and drafting of the manuscript. ALS conceived of the study and its design and drafted the manuscript. All authors read and approved the final manuscript.
